# Multimodal prediction based on ultrasound for response to neoadjuvant chemotherapy in triple negative breast cancer

**DOI:** 10.1038/s41698-025-01057-7

**Published:** 2025-07-25

**Authors:** Maohua Lyu, Shouheng Yi, Chunyan Li, Yu Xie, Yu Liu, Zeyan Xu, Zhitao Wei, Huan Lin, Yunlin Zheng, Chunwang Huang, Xi Lin, Zaiyi Liu, Shufang Pei, Biao Huang, Zhenwei Shi

**Affiliations:** 1 Department of Radiology, Guangdong Provincial People’s Hospital (Guangdong Academy of Medical Sciences), Southern Medical University, Guangzhou, China; 2 Guangdong Provincial Key Laboratory of Artificial Intelligence in Medical Image Analysis and Application, Guangzhou, China; 3 School of Electronic Engineering, Xi’an Shiyou University, Xi’an, China; 4 Department of Ultrasound, State Key Laboratory of Oncology in South China, Guangdong Provincial Clinical Research Center for Cancer, Sun Yat-sen University Cancer Center, Guangzhou, China; 5 Department of Radiology, Yunnan Cancer Hospital, The Third Affiliated Hospital of Kunming Medical University, Peking University Cancer Hospital, Yunnan, China; 6 Department of Ultrasound, Guangdong Provincial People’s Hospital (Guangdong Academy of Medical Sciences), Southern Medical University, Guangzhou, China; 7 Institution of Computational Science and Technology, Guangzhou University, Guangzhou, China; 8 Xinjiang Key Laboratory of Artificial Intelligence Assisted Imaging Diagnosis, Kashi, China

**Keywords:** Breast cancer, Surgical oncology, Breast cancer, Predictive markers

## Abstract

Pathological complete response (pCR) can guide surgical strategy and postoperative treatments in triple-negative breast cancer (TNBC). In this study, we developed a Breast Cancer Response Prediction (BCRP) model to predict the pCR in patients with TNBC. The BCRP model integrated multi-dimensional longitudinal quantitative imaging features, clinical factors and features from the Breast Imaging Data and Reporting System (BI-RADS). Multi-dimensional longitudinal quantitative imaging features, including deep learning features and radiomics features, were extracted from multiview B-mode and colour Doppler ultrasound images before and after treatment. The BCRP model achieved the areas under the receiver operating curves (AUCs) of 0.94 [95% confidence interval (CI), 0.91–0.98] and 0.84 [95%CI, 0.75–0.92] in the training and external test cohorts, respectively. Additionally, the low BCRP score was an independent risk factor for event-free survival (*P* < 0.05). The BCRP model showed a promising ability in predicting response to neoadjuvant chemotherapy in TNBC, and could provide valuable information for survival.

## Introduction

Breast cancer is the malignancy with the highest incidence of morbidity and mortality in women worldwide^1^. Triple-negative breast cancer (TNBC) subtype represents 15% of all breast cancer, and has the worst prognosis due to a lack of targeted therapies^2,3^. Neoadjuvant chemotherapy (NACT) is a frequently utilized treatment modality for non-metastatic TNBC. Unfortunately, the responses to NACT vary considerably among patients, with some exhibiting positive outcomes while others show poor responses^4^.

Pathologic complete response (pCR) is commonly utilized as a metric for assessing the effectiveness of NACT and has been confirmed as a surrogate endpoint for long-term prognosis in breast cancer^5^. In comparison to the other subtypes, the pCR of TNBC exhibited the most robust correlation with prognosis^6^. Patients diagnosed with TNBC who achieve pCR may benefit from reduced requirements for extensive breast surgery and decreased reliance on intensive postoperative adjuvant therapies^7,8^. Nevertheless, the confirmation of pCR requires surgical pathology evaluation, leading to a delay. Unfortunately, currently, few established biomarkers are available for predicting pCR preoperatively^9^. Hence, there is an urgent demand in clinical practice for preoperative methods to assess pCR, which can assist in clinical management and decision-making.

Ultrasound (US) imaging serves as the most commonly used imaging tool for monitoring the efficacy of NACT in clinical practice. Compared with X-ray and MRI, US imaging has advantages in monitoring treatment response due to its radiation-free, rapid, and reproducible nature. US images can capture data pertinent to treatment response; yet, the visual information they provide is limited^10^, and the interpretation of these images by humans is inherently subjective. To address these challenges, high-throughput information extracted from US images through machine learning techniques, including radiomics and deep learning, has demonstrated significant promise in predicting the effectiveness of NACT in breast cancer^11–15^. Nevertheless, previous studies have been constrained by certain limitations. First, previous studies have not fully utilized US image information. They frequently rely on a single B-mode US image, not considering multiview or multimodal US images in consideration^11–13^. Second, previous research does not consider Breast Imaging Data and Reporting System (BI-RADS) features^11–15^. Finally, previous researches lack prediction models of the treatment response specifically for TNBC subtype^11–15^, and limited data from a single center impedes the research on TNBC^14,15^. The aforementioned challenges emphasize the need for additional research.

In this study, we aimed to integrate multimodal US-based information to enhance the performance of the predictive model. We employed machine learning techniques to thoroughly analyze longitudinal US information using multiview B-mode and color Doppler images. Subsequently, we integrated the image data with clinical factors and BI-RADS features to develop and validate a Breast Cancer Response Prediction (BCRP) model.

## Results

### Patient characteristics and BI-RADS information

A total of 283 female patients provided a total of 1698 B-mode and color Doppler images for inclusion in the study (Supplementary Fig. 1). In the training cohort (TC), there were 173 patients; 91 patients (52.6%) achieved pCR. The external testing cohort (ETC) comprised 110 patients; 35 patients (31.8%) achieved pCR. The mean age of the TC was 49.0 (standard deviation: 10.4; range: 23.0 to 79.0) years, while the ETC was 46.7 (standard deviation: 8.6; range: 27.0–69.0) years. The majority of lesions exhibited characteristics consistent with T2 invasive ductal carcinoma, accompanied by lymph node metastasis and high Ki67 expression (>30%)^16^. The primary treatment involved Anthracycline and Taxane drugs for at least six cycles.

It is worth noting that the ETC comprised a greater percentage of young premenopausal women with elevated *T* and *N* stages, and the masses in TC were more likely to exhibit calcification and posterior echo enhancement. The detailed clinical baseline characteristics of the patients and the BI-RADS characteristics of the lesions were outlined in Tables 1 and 2, respectively.

**Table 1:** Baseline patient clinical characteristics

Clinical characteristics	TC (n = 173) n (%)	ETC (n = 110) n (%)	*P* value
Age (years)			0.046*
Mean ± SD	49.0 ± 10.4	46.7 ± 8.6	
Range	(23.0–79.0)	(27.0–69.0)	
Menopause			0.049*
Premenopausal	96 (55.5)	74 (67.3)	
Postmenopausal	77 (44.5)	36 (32.7)	
T stage			0.002*
T1	16 (9.2)	7 (6.4)	
T2	130 (75.1)	68 (62.4)	
T3	15 (8.7)	17 (15.6)	
T4	12 (6.9)	17 (15.6)	
NA	0 (0.0)	1 (0.9)	
N stage			<0.001*
N0	71 (41.0)	16 (14.7)	
N1	70 (40.5)	49 (45.0)	
N2	26 (15.0)	25 (22.9)	
N3	6 (3.5)	29 (17.4)	
NA	0 (0.0)	1 (0.9)	
Histologic type			0.500
IDC	160 (92.5)	104 (94.5)	
Others	13 (7.5)	6 (5.5)	
Ki67			0.109
<30%	21 (12.1)	19 (18.1)	
≥30%	152 (87.9)	86 (81.9)	
NA	0 (0.0)	5 (4.5)	
NACT regimen			<0.001*
Anthracycline-based	4 (2.3)	11 (10.1)	
Taxane-based	74 (42.8)	7 (6.4)	
Anthracycline and Taxane-based	95 (54.9)	91 (83.5)	
NA	0 (0.0)	1 (0.9)	
Platinum			0.296
No	141 (81.5)	84 (76.4)	
Yes	32 (18.5)	26 (23.6)	
NACT cycle			0.437
< 6	24 (13.9)	19 (17.3)	
≥ 6	149 (86.1)	91 (82.7)	

*P* values were calculated using the chi-square test, Fisher’s exact test, or Mann–Whitney U test. An asterisk indicates a significant *P* value. *IDC* invasive ductal carcinoma, *NACT* neoadjuvant chemotherapy.

**Table 2:** Baseline patient BI-RADS characteristics

BI-RADS characteristics	TC (n = 173) n (%)	ETC (n = 110) n (%)	*P* value
Shape			0.004*
Oval or round	71 (41.0)	27 (24.5)	
Irregular	102 (59.0)	83 (75.5)	
Orientation			0.081
Parallel	127 (73.4)	70 (63.6)	
Not parallel	46 (26.6)	40 (36.4)	
Margin			<0.001*
Circumscribed	90 (52.0)	28 (25.5)	
Not circumscribed	83 (48.0)	82 (74.5)	
Echo pattern			0.012*
Complex cystic-solid	6 (3.5)	14 (12.7)	
Hypoechoic	120 (69.4)	71 (64.5)	
Heterogeneous	47 (27.2)	25 (22.7)	
Posterior feature			0.002*
No posterior feature	28 (16.2)	31 (28.2)	
Enhancement	99 (57.2)	56 (50.9)	
Shadowing	28 (16.2)	22 (20.0)	
Combined pattern	18 (10.4)	1 (0.9)	
Calcification			0.001*
No	71 (41.0)	68 (61.8)	
Yes	102 (59.0)	42 (38.2)	
Vascularity			0.188
Absent	16 (9.2)	5 (4.5)	
Internal vascularity	44 (25.4)	36 (32.7)	
Vessels in rim	113 (65.3)	69 (62.7)	
Architectural distortion			0.146
No	13 (7.5)	14 (12.7)	
Yes	160 (92.5)	96 (87.3)	
Duct change			0.715
No	169 (97.7)	106 (96.4)	
Yes	4 (2.3)	4 (3.6)	
Skin change			0.560
No	162 (93.6)	101 (91.8)	
Yes	11 (6.4)	9 (8.2)	
Edema			0.771
No	165 (95.4)	106 (96.4)	
Yes	8 (4.6)	4 (3.6)	

*P* values were calculated using the chi-square test or Fisher’s exact test. An asterisk indicates a significant *P* value. *BI-RADS* breast imaging data and reporting system.

### Performance of models based on longitudinal US images

A total of 24 delta deep learning features were incorporated into the construction of deep learning (DL) model, achieving the area under the receiver operating curve (AUC) values of 0.88 [95% confidence interval (CI), 0.83–0.93] and 0.77 [95%CI, 0.66–0.87] in the TC and ETC, respectively.

The radiomics (Rad) model was developed utilizing radiomics features, achieved AUC values of 0.84 [95%CI, 0.78–0.90] and 0.72 [95%CI, 0.62–0.83] in the TC and ETC, respectively. Additional details can be found in Supplementary Table 1.

The Image model was developed through the integration of the Rad scores and DL scores, leading to improved performance as evidenced by AUCs of 0.90 [95%CI, 0.85–0.95] for TC and 0.80 [95%CI, 0.71–0.90] for ETC. Generally, the Image model showed better predictive ability than either DL or Rad models in both cohorts (Supplementary Table 2). The performance of each model was detailed in Table 3. Furthermore, the selected deep learning features had weak correlation with the radiomics features, indicating they were not redundant but complementary (Supplementary Fig. 2).

**Table 3:** Performance of models in predicting pathological complete response

	DL model	Rad model	Clinic model	BR model	Image model	BCRP model
**TC**						
AUC	0.88 (0.83, 0.93)	0.84 (0.78, 0.90)	0.66 (0.58, 0.74)	0.63 (0.54, 0.71)	0.90 (0.86, 0.95)	0.94 (0.91, 0.98)
Accuracy	0.83 (0.77, 0.89)	0.78 (0.71, 0.85)	0.64 (0.56, 0.72)	0.61 (0.52, 0.69)	0.83 (0.77, 0.89)	0.86 (0.81, 0.92)
Sensitivity	0.81 (0.75, 0.88)	0.76 (0.69, 0.83)	0.82 (0.76, 0.89)	0.69 (0.61, 0.77)	0.76 (0.69, 0.83)	0.80 (0.74, 0.87)
Specificity	0.84 (0.78, 0.90)	0.80 (0.74, 0.87)	0.44 (0.35, 0.52)	0.51 (0.42, 0.60)	0.90 (0.86, 0.95)	0.93 (0.89, 0.97)
PPV	0.85 (0.79, 0.91)	0.81 (0.75, 0.88)	0.62 (0.54, 0.70)	0.61 (0.52, 0.70)	0.90 (0.85, 0.94)	0.92 (0.88, 0.97)
NPV	0.80 (0.74, 0.87)	0.75 (0.68, 0.82)	0.69 (0.61, 0.77)	0.60 (0.52, 0.68)	0.77 (0.70, 0.84)	0.81 (0.74, 0.88)
**ETC**						
AUC	0.77 (0.66, 0.87)	0.72 (0.62, 0.83)	0.55 (0.43, 0.66)	0.58 (0.46, 0.69)	0.80 (0.71, 0.90)	0.84 (0.75, 0.92)
Accuracy	0.71 (0.60, 0.82)	0.73 (0.62, 0.83)	0.51 (0.39, 0.63)	0.55 (0.43, 0.66)	0.75 (0.64, 0.85)	0.77 (0.67, 0.87)
Sensitivity	0.71 (0.61, 0.82)	0.74 (0.64, 0.85)	0.49 (0.37, 0.60)	0.60 (0.49, 0.72)	0.63 (0.51, 0.74)	0.74 (0.64, 0.85)
Specificity	0.71 (0.60, 0.82)	0.72 (0.61, 0.83)	0.52 (0.40, 0.64)	0.52 (0.40, 0.64)	0.80 (0.70, 0.90)	0.79 (0.69, 0.89)
PPV	0.53 (0.42, 0.65)	0.55 (0.44, 0.67)	0.32 (0.22, 0.42)	0.37 (0.26, 0.48)	0.59 (0.48, 0.71)	0.62 (0.50, 0.73)
NPV	0.84 (0.75, 0.93)	0.86 (0.77, 0.94)	0.68 (0.57, 0.80)	0.74 (0.63, 0.84)	0.82 (0.73, 0.91)	0.87 (0.79, 0.95)

Data are presented as mean with 95% confidence interval. *AUC* area under the receiver operating characteristic curve, *PPV* positive predictive value, *NPV* negative predictive value.

### Performance of the clinic and BR models

The Clinic model finally incorporated two clinical factors, N stage and Ki67, achieving AUCs of 0.66 [95%CI, 0.58–0.74] and 0.55 [95%CI, 0.43–0.66] in the TC and ETC, respectively. The incorporation of the two factors of posterior feature and calcification into the BI-RADS model (BR) had AUCs of 0.63 [95%CI, 0.54–0.71] and 0.58 [95%CI, 0.46–0.69] in the TC and ETC, respectively. The evaluation of interobserver agreement revealed a substantial level of agreement for edema (kappa = 0.70) and shape (kappa = 0.75), while for the remaining BI-RADS features, the agreement was observed to be almost perfect (kappa > 0.80).

### Performance of the BCRP model

**Fig. 1:**
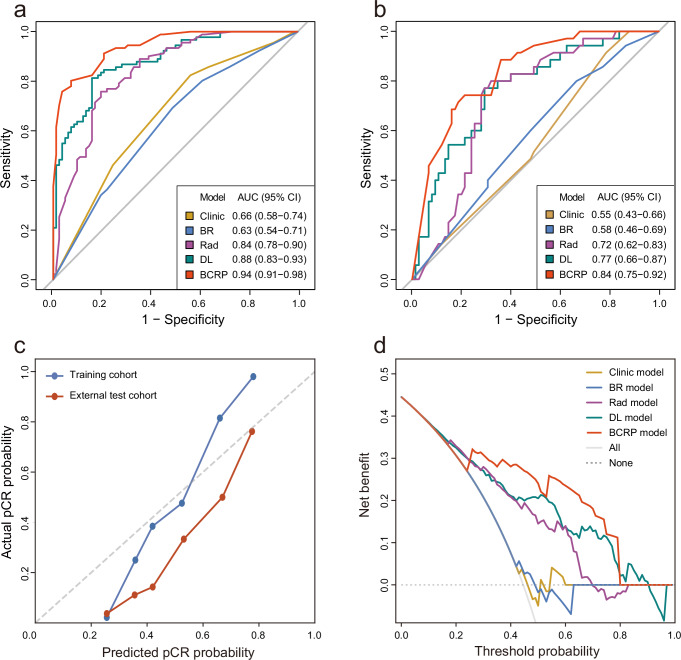
Performance of models in pCR prediction. Receiver operator characteristic curves of DL, Rad, Clinic, BR, and BCRP models for predicting pCR in the **a** training cohort and **b** external test cohort. **c** Calibration curves of BCRP model in the training and external test cohort. **d** Decision curve analysis for DL, Rad, Clinic, BR, and BCRP models. *pCR* pathological complete response, *AUC* areas under the receiver operating curve.

The BCRP model, which incorporated the predictions of DL, Rad, Clinic, and BR models, demonstrated an AUC of 0.94 [95%CI, 0.91–0.98] and 0.84 [95%CI, 0.75–0.92] in the TC and ETC, respectively. As shown in Fig. 1a, b, these values exhibited a statistically significant increase compared to any individual model within both TC and ETC (*P* < 0.05). Figure 1c illustrated a significant agreement between the predicted pCR as determined by the BCRP model and the observed outcomes in both TC and ETC (*P* > 0.05), the Brier scores were 0.13 and 0.19. Figure 1d demonstrated that BCRP produces greater net gains compared to other models over the relevant threshold range among the entire cohort.

**Fig. 2:**
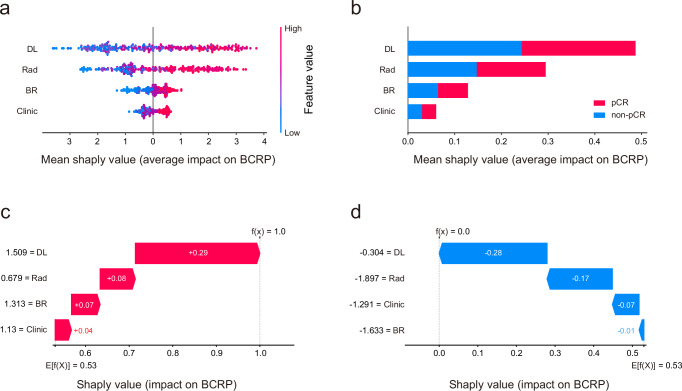
Shapley values for the interpretation of BCRP. The scores with larger shaply values are considered to be more important in BCRP decision-making. **a** Shaply summary dot plot on all cohort. **b** Shaply summary bar plot on all cohort. **c** A patient with pCR. **d** A patient with non-pCR. *pCR* pathological complete response.

In the TC, the optimal threshold of BCRP scores was determined to be 0.55 based on the maximum Youden index. DL scores played a pivotal role in BCRP’s decision-making process, with Rad scores being of secondary importance, followed by BR and Clinic scores (Fig. 2a, b). Local interpretation examined the mechanisms by which the BCRP model generated predictions for individual cases. Figure 2c, d illustrated the BCRP decisions for a patient achieving pCR and non-pCR, respectively, both of which were accurately predicted. As illustrated in Fig. 2c, the values of the four scores pushed decision towards pCR. Conversely, as depicted in Fig. 2d, the overall values pushed decision towards non-pCR. In addition, we defined a high sensitivity threshold of 0.33. In the TC and ETC, this threshold achieved sensitivities of 98.9 and 97.1%, respectively. The model’s performance under the high sensitivity threshold is listed in Supplementary Table 3. The confusion matrices under different thresholds can be seen in Supplementary Fig. 3.

**Fig. 3:**
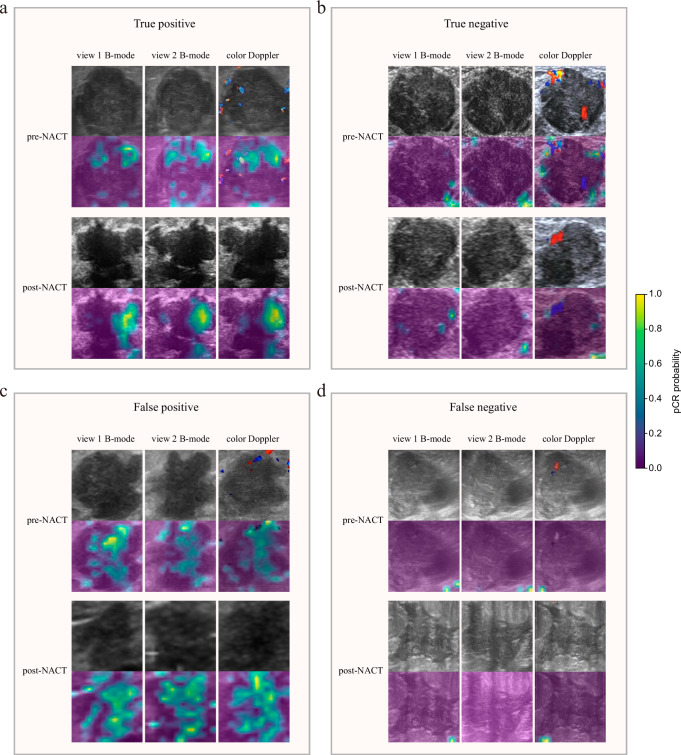
Visualization and interpretation of the DL model for pCR prediction. Activation maps from pre- and post-NACT images for **a** true positive, **b** true negative, **c** false positive, and **d** false negative patients are displayed. The colors of the superimposed heat maps denote the predicted probability of achieving pCR. *pCR* pathological complete response, *NACT* neoadjuvant chemotherapy.

Upon employing heatmaps to visualize the decision-making process of the DL model, it was observed that the US image data within the lesion significantly contributed to the prediction of pCR (Fig. 3a, c). Conversely, for non-pCR patients, the model was unable to extract effective predictive information from within the tumor (Fig. 3b, d).

### Preoperative predictors of survival

**Fig. 4:**
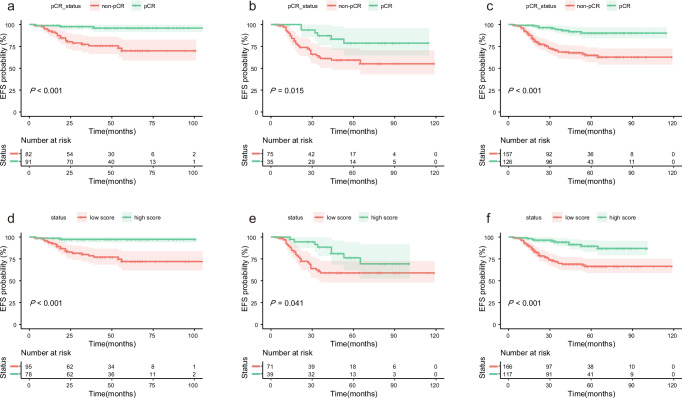
Kaplan-Meier curves of EFS on total cohorts. Kaplan-Meier curves of EFS between actual pCR and non-pCR in **a** training cohort **b** external test cohort, and **c** entire cohort. Kaplan-Meier curves of EFS between high and low BCRP scores based on cutoff valve of 0.59 in **d** training cohort **e** external test cohort, and **f** entire cohort. *EFS* event-free survival, *pCR* pathological complete response.

The follow-up of patients was finished at 22 June 2024, and the median follow-up time was 51 (interquartile range: 34–68) months. Maximally selected log-rank statistics were employed to ascertain the optimal cutoff value of 0.59, and all patients were categorized into low and high score groups. Compared with low-score patients, the high-score groups had a better EFS (*P* < 0.05), as shown in Fig. 4. The BCRP scores were found to be an independent risk factor for event-free survival (EFS) in both univariate and multivariate Cox regression analyses (Supplementary Table 4). Using independent risk factors, the final Cox regression model demonstrated a C index of 0.75 [95%CI, 0.68–0.81].

## Discussion

In this study, we collected TNBC cases from multiple medical centers. Each patient underwent examination using multiview B-mode and color Doppler US imaging, both pre- and post-NACT. We developed and validated a predictive model (BCRP), which combined quantitative US imaging features based on imaging, radiologist’ assessment of image features, and baseline clinical parameters. The BCRP consistently outperformed the DL, Rad, Clinic, and BR models, both in TC and ETC. This highlighted the superiority of integrating multimodal information over depending on unimodality for evaluating the response to NACT, which is in line with previous studies^17,18^. It is noteworthy that BCRP scores exhibited a significant correlation with EFS and facilitated the stratification of patient prognosis.

Of the 126 patients with pCR, BCRP successfully identified 99 (78.6%) who might benefit from relieving surgery and postoperative treatment. However, 27/126 (21.4%) patients were incorrectly predicted as non-pCR, and 22/157 (14.0%) patients were incorrectly predicted to be pCR. Previous studies have shown that vacuum-assisted core biopsy (VACB) demonstrated a sensitivity of 90% and specificity of 100% in detecting residual disease in the breast^19^, and patients achieving a pCR in the breast exhibited a very low risk (4.1%) of axillary metastasis^20^. For patients predicted to be non-pCR, the use of VACB might be avoided due to its invasive nature, as 83.3% of these patients did not require VACB. Even though this approach would result in 21.4% of pCR patients being incorrectly predicted as non-pCR, overtreatment could not be avoided. 14.0% patients were wrongly predicted to be pCR. If the treatment of these patients is reduced, it may result in a poor prognosis, which is unacceptable. Therefore, for patients predicted to be pCR, VACB might be used as a supplementary examination to accurately identify non-pCR patients and prevent undertreatment. In order to enhance the clinical utility, we defined a high sensitivity threshold for the BCRP model. Patients with BCRP scores below the threshold, 73/75 (97.3%), could potentially benefit from more stringent surgical procedures and enhanced follow-up to prevent undertreatment. In comparison, VACB accurately identified 19/21 (90.5%) patients with residual breast lesion^19^, showing inferior performance compared to the BCRP model. On the other hand, for patients with BCRP scores exceeding the threshold, further VACB evaluation could be a valuable tool to precisely ascertain the response to NACT, despite the invasiveness of VACB.

After an extensive screening of numerous features, we determined that the post-treatment and delta features within the radiomics domain, as well as the delta features within the deep learning domain, were most efficacious in predicting response to treatment. This indicated that post-treatment and delta features may offer more valuable insights into treatment effectiveness compared to pre-treatment features, which is consistent with previous studies^21^. Furthermore, the features derived from a combination of multiview B-mode and color Doppler images offer valuable insights for predicting pCR in BCRP model. This finding underscored the notion that multimodal multiview US images yield more significant information about lesions than a single B-mode US image^22,23^.

In our study, the DL predictions were the most important factor of BCRP model. The pre-training process compels model to acquire a robust representation of image features^24^. Previous research predominantly utilized natural images from the ImageNet dataset for pre-training purposes^25,26^. In contrast, the self-pretraining approach demonstrates notable advantages, particularly in situations where acquiring pre-training data is challenging^27^. Based on the Masked Autoencoders (MAE) framework, which incorporates an efficient global self-attention mechanism^28^, our DL model was pre-trained utilizing US imaging data. Unlike conventional radiomics approaches, which focus exclusively on the subtle features within the tumor, deep learning models have the capability to incorporate peritumoral tissues. Our study illustrated that deep learning and radiomics features could synergistically enhance each other in terms of informational content.

After NACT, the residual lesions shown on imaging may contain tumor parenchymal cells and fibrous stroma retained in various forms^29^. Radiologists cannot accurately determine pCR status based on US images^30^. The excellent performance of the BCRP model mainly benefits from the DL features; however, the DL features are inherently poorly interpretable, and these gradient weighted class activation mapping (Grad-CAM) cannot be easily deciphered by humans^11^. We try to visualize the DL features from the perspective of individual cases. It is known that the features included in the DL model are post-NACT features and delta features. We believe that the information used by the DL model to predict pCR comes from within the tumor and is closely related to the changes in intratumoral echoes. The information that the model predicts as pCR (Fig.3a, c) mainly comes from the lower echo area inside the tumor. The existence of lower echo area after treatment and the resulting grayscale and texture changes before and after treatment may be the reasons why the model predicts pCR. In contrast, the model predicted non-pCR (Fig.3b, d) because the overall echogenicity of the tumor after treatment was high and the echo components were mixed. The model did not capture these parts of the tumor images as information about whether the patient had achieved pCR. In addition, we found that the tumor shape of patients who achieved pCR (Fig.3a, d) changed significantly after treatment and was more irregular than that of non-pCR patients (Fig.3b, c). Interestingly, previous studies have shown that increased internal echogenicity of the tumor after NACT^31^ and regularized morphology^32^ can be used as indicators of good NACT efficacy. This may be because other studies did not analyze TNBC but all subtypes of breast cancer. The biological significance behind these findings deserves further exploration.

In addition, clinical and BI-RADS features further improved the effectiveness of the BCRP model. The features of BI-RADS, which are acknowledged and standardized terminologies, are employed by radiologists for the evaluation of breast lesion characteristics, yet their assessment is inherently subjective. Our research demonstrated that the inter-reader agreement for the assessment of BI-RADS features was satisfactory, following pre-assessment communication.

Ogier du Terrail J et al^33^. included 656 TNBC patients, the largest sample size to date, and built a multimodal federated learning model based on whole-slide imaging slices, with AUCs of less than 0.8 for predicting pCR. Y. Zhang et al^34^. included 112 TNBC patients based on longitudinal MRI images (pre-NACT and after two cycles), as well as genetic information, their model achieved an AUC of 0.87 in the absence of external test datasets. Outperforming these models, the BCRP achieved the best results of 0.94 and 0.84 in the TC and ETC. Longitudinal US images have the capability to capture the dynamic characteristics of the entire tumor region, showcasing its evolving features throughout the treatment process^35–37^; therefore, it is speculated that longitudinal multimodal US images may provide more comprehensive information compared to whole-slide imaging obtained through puncture for the purpose of predicting treatment response. In addition, the advantage of our model is that the input is US images, which are non-invasive, cheap and easy to obtain, so our model has higher clinical practicality. It is worth noting that Yu Liu and colleagues^12^ used deep learning features from longitudinal US images to predict pCR in HER2-positive breast cancer, achieving AUCs over 0.90. This disparity in performance might indicate distinctive unique challenges in TNBC.

There are also several limitations to the present study. First, retrospective designs are prone to selection bias. However, conducting a randomized, controlled trial with TNBC patients who received NACT was extremely difficult, given the limited patients of this tumor subtype. Second, it should be noted that the sample size of this study, while it may meet the minimum requirement for sample size assessment, is relatively small. The small sample size will undoubtedly lead to a lack of case diversity, and therefore, the model may fail to capture deeper image information to obtain better predictive performance. The results need to be confirmed by expanding the sample size, even if our model achieved excellent performance in the TC and ETC. Third, magnetic resonance imaging and genetic data have shown considerable potential in forecasting treatment outcomes^34,38–40^. Nonetheless, these modalities were not incorporated in our research due to constraints in available data. The possibility of integrating such information warrants further exploration in future studies.

In conclusion, BCRP model incorporated data from US images, clinical factors, and BI-RADS features, leading to superior performance in both TC and ETC. Our research underscores the imperative of incorporating multimodal US-based data to enhance the precision of predicting responses to NACT, particularly for TNBC subtype, where prediction poses greater challenges. Further research, refinement, and validation of the BCRP model is necessary to enhance its utility in clinical decision-making.

## Methods

This multicenter study was conducted in accordance with the Declaration of Helsinki and approved by the institutional review boards of Guangdong Provincial People’s Hospital (GPPH), Sun Yat-sen University Cancer Center (SYSUCC) and Yunnan Cancer Hospital (YNCH). Informed consent was waived due to the retrospective and non-invasive nature of the study. The report was prepared in accordance with the TRIPOD + AI statement^41^.

### Study design and participants

**Fig. 5:**
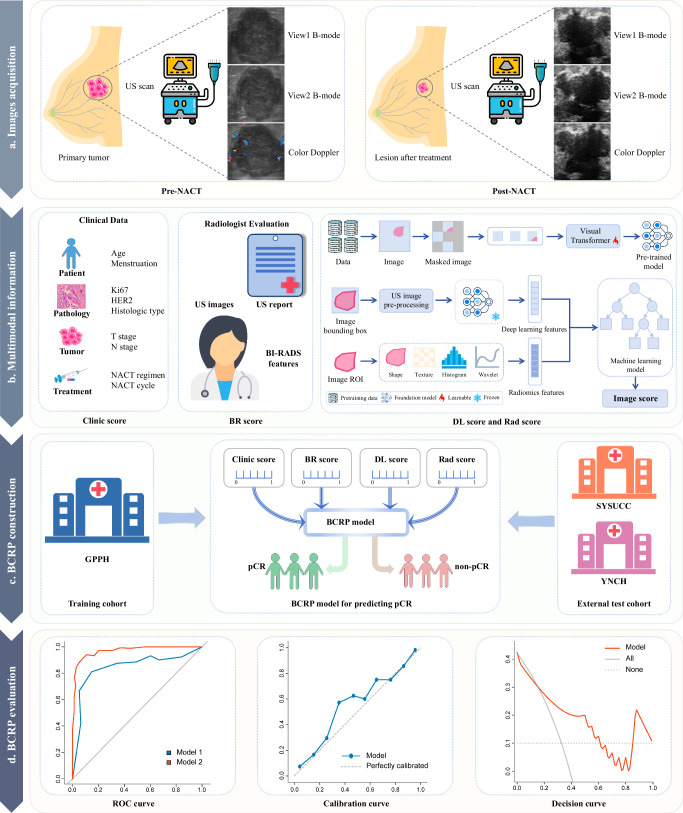
Flowchart of this study. **a** US images acquisition. **b** Multimodal information. **c** Development and validation of the BCRP model. **d** Performance evaluation of BCRP model. *BI-RADS* breast imaging data and reporting system, *US* ultrasound, *NACT* neoadjuvant chemotherapy, *GPPH* Guangdong Provincial People’s Hospital, *SYSUCC* Sun Yat-sen University Cancer Center, *YNCH* Yunnan Cancer Hospital.

This study consecutively enrolled 283 TNBC patients without distant metastasis from three hospitals. The TC consisting of 1038 images from 173 patients recruited from GPPH. The ETC comprising 660 images from 110 patients treated at SYSUCC and YNCH, considering sample size requirement. Following Anthracycline and Taxane-based NACT treatment, all patients underwent breast and axillary surgery. Baseline clinical data were retrieved from the patients’ medical records. The study design followed Fig. 5.

### Image collection and BI-RADS assessment

All patients underwent a systematic US examination at two time points: one week before NACT initiation and two weeks preoperative following completion of NACT. Examinations were mainly performed using General Electric (LOGIQ E9), Hitachi (Ascendus), Toshiba (Aplio 500) or Mindray (DC-80) with a 4–15 MHz linear transducer. The collected US images were converted into JPEG format in GPPH and SYSUCC, while those were converted into DICOM format in YNCH. All US scans were obtained from the breast imaging database.

For each patient, a total of six images were chosen, including two B-mode US images and one color Doppler image, both pre- and post-treatment. Delineations of the region of interest (ROI) were performed using ITK-SNAP 4.0 after converting all images to NIFTI format. Specifically, if conventional tumor lesions were not observed following NACT, the ROI was defined using the fibrotic tissue in the area where the tumor had previously been located^11^.

According to BI-RADS features published by the American College of Radiology^42^, breast lesion characteristics observed in US images are categorized into 11 distinct groups. The BI-RADS assessment relies solely on the US images and corresponding report findings, while other information was blind. The US images were screened, delineated, and evaluated using the BI-RADS feature by radiologist 1 with 6 years of experience in breast US examination. After three months, a random sample of 60 patients was selected from TC to perform a re-delineation of ROIs. This process was conducted by radiologist 1 and radiologist 2 (with 6 years of experience). Before conducting the BI-RADS evaluation, radiologist 1 accrued substantial experience through the evaluation of an additional 800 breast cancer patients, independent of this study. Radiologist 2 conducted BI-RADS evaluations on a sample of 60 patients selected at random. Prior to this evaluation, the two radiologists discussed and evaluated the US images of another ten typical lesion based on the BI-RADS criteria and reached a consensus.

### Definition of TNBC and outcomes

TNBC is defined by the absence of expression of estrogen receptor (ER), progesterone receptor (PR), and human epidermal growth factor receptor 2 (HER2), where we defined absence of ER/PR expression as less than 10% expression^43,44^, HER2-negative was characterized by immunohistochemistry (IHC) 0 or IHC 1 + , or IHC 2+ with negative results in fluorescence in situ hybridization. The status of pCR was operationally defined as the lack of invasive residual tumor cells in both breast and axillary lymph nodes^6^. EFS was determined as the duration from the initiation of NACT to the occurrence of the first event, which encompassed invasive local, regional, and distant recurrences; contralateral breast cancer; second non-breast primary cancer; and all-cause mortality, with the exception of carcinoma in situ of the breast^45^.

### Image preprocessing

Initially, the raw images were cropped to remove surrounding black borders, icons and textual information. Subsequently, the OpenCV toolkit was utilized to resize the images uniformly to 224 × 224 pixels via bilinear interpolation. For the ground truth annotations, a nearest neighbor method was employed for size scaling. All longitudinal US images were resampled to a uniform spatial resolution and subsequently subjected to z-score normalization. During the pre-training process, all images were augmented to expand the dataset and help alleviate the overfitting. The employed augmentation techniques encompassed random horizontal and vertical flips, random cropping (with the cropped area varying between 20 and 100% of the original image size), and subsequent resizing using bicubic interpolation.

### Construction of DL, Rad, clinic, BR, and BCRP models

A pre-training approach based on the MAE architecture was adopted^24^. During the pre-training process, the MAE randomly occluded 75% of the image pixels, compelling the network to learn high-level semantic features from the remaining visible pixels to reconstruct the full images. This approach can proficiently capture and comprehend the global characteristics inherent in the images. The MAE was pre-trained using US images without annotations to ensure that the model captures rich visual information. The AdamW optimizer was employed for the pre-training phase of the DL model. After pre-training, the encoder part of the MAE was used as a feature extractor to extract deep learning features from the bounding box regions of the images.

Radiomics features were extracted from tumor regions using the PyRadiomics package (version 3.0.1). The features included two major categories: Original, which encompassed features from the original image, and Wavelet, which involved features derived from wavelet transforms. These features covered histogram-based intensity, shape, and various gray-level matrices.

Following the above steps, the relative change values of deep learning and radiomics features before and after treatment were computed and designated as delta features. A total of 6912 deep learning features and 2637 radiomics features were obtained. To guarantee the robustness of the radiomics features, we retained features with intra- and inter-class correlation coefficients greater than 0.75. The Pearson correlation coefficient, analysis of variance, and the Mann-Whitney U-test were utilized to determine the most representative features through a systematic screening process. Finally, 24 deep learning and 14 radiomics features were selected. And then, we employed the XGBoost algorithm to construct the DL model and the Rad model independently. Furthermore, utilizing the DL scores and Rad scores, the Image model was also developed using the XGBoost algorithm.

In the TC, univariate logistic regression was utilized to evaluate the association of clinical and BI-RADS parameters with pCR. Multivariate logistic regression was adopted to develop Clinic and BR models based on factors that demonstrated statistical significance in univariate analysis (*P* < 0.1). The prediction scores of Rad, DL, Clinic, and BR were utilized as variables in the development of BCRP model using the XGBoost algorithm. The optimal hyperparameters were determined through grid search during the model development process.

### Model evaluation

Multiple statistics were employed to measure model performance, including AUC, accuracy, sensitivity, specificity, positive predictive value (PPV), and negative predictive value (NPV). To evaluate the calibration performance and clinical utility, a calibration curve and a decision curve were generated. Model calibration was measured using the Brier score. The DeLong test, integrated discrimination improvement (IDI), and net reclassification improvement (NRI) were employed to assess the efficacy of various models in identifying pCR.

To improve the transparency of BCRP model decisions, Shapley values were employed to quantify the individual contributions of the factors incorporated into the model. Grad-CAM was employed to identify attentional regions in order to improve the interpretability of the DL model. Furthermore, the relationship between BCRP scores and EFS was evaluated through the utilization of Kaplan-Meier curves and the log-rank test.

### Statistical analysis

The Mann-Whitney U test was employed for continuous and ordered categorical variables, while the chi-square test or Fisher’s exact test was utilized for unordered categorical variables to assess group comparisons. Kappa statistics were employed to evaluate interobserver agreement of BI-RADS characteristics, with 0.6–0.8 indicating substantial agreement and >0.8 representing almost perfect agreement^46^. Kaplan-Meier survival curves and the log-rank test were employed to analyze and compare the EFS. With multiple imputations, the missing values of clinical baseline parameters were imputed^47,48^. To achieve a statistical power of 90%, a minimum sample size of 64 patients is needed for the external test set, based on an AUC of 0.50 for the null hypothesis and 0.75 for the alternative hypothesis, with a 30% proportion of pCR to non-pCR patients^49^. The statistical analysis was conducted using Python (version 3.10.13), SPSS (version 26.0), R (version 4.4.0), and MedCalc (version 20.027) software. Statistical significance was determined by two-sided *P* < 0.05, unless otherwise indicated.

## Supplementary information

Supplementary materials

## Data Availability

The data and code for analysis in this work are available to researchers upon reasonable request. Please e-mail the corresponding author, Zhenwei Shi, Ph.D., at shizhenwei@gdph.org.cn.

## Code availability

The code utilized in this research has not been publicly disclosed, however, it can be accessed by qualified researchers upon a reasonable request. Please e-mail the corresponding author, Zhenwei Shi, Ph.D., at shizhenwei@gdph.org.cn.
