# Supplementary material for: Multimodal prediction based on ultrasound for response to neoadjuvant chemotherapy in triple negative breast cancer
Source: NPJ Precis Oncol. 2025 Jul 25;9:259. doi: 10.1038/s41698-025-01057-7 (PMC12297240; doi:10.1038/s41698-025-01057-7)
Supplement: Supplementary file 1 — Supplementary materials [file 41698_2025_1057_MOESM1_ESM.pdf]

Supplementary materials

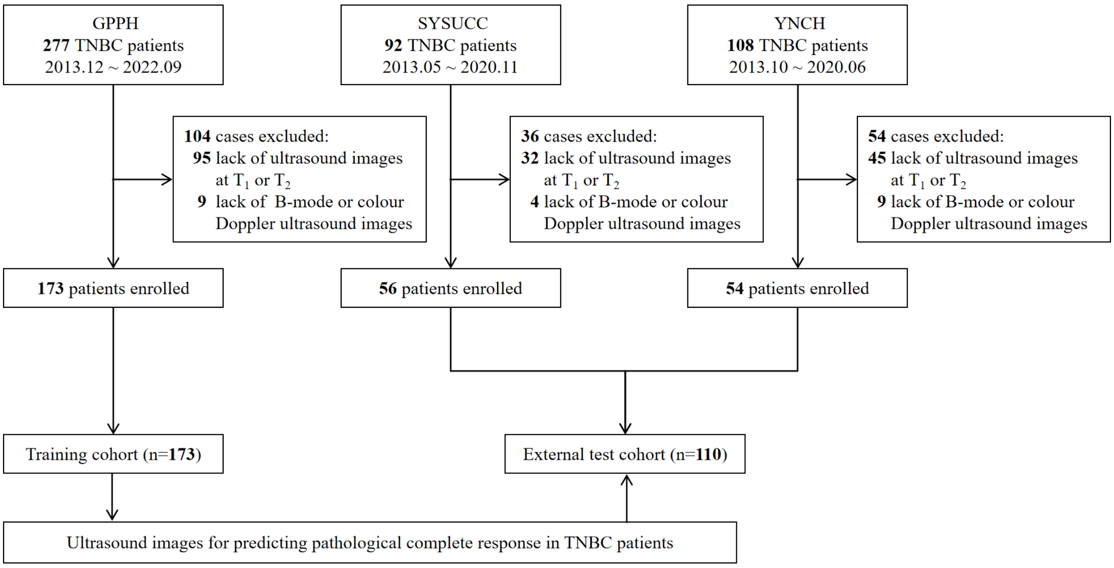

Supplementary Figure 1. Flowchart of study participants. 283 patients with TNBC from three centers were included in the study, each providing two views B-mode and one colour Doppler ultrasound images before and after NACT treatment. *TNBC* triple-negative breast cancer, *NACT* neoadjuvant chemotherapy, *T1* pre-NACT, *T2* post-NACT, *GPPH* Guangdong Provincial People's Hospital, *SYSUCC* Sun Yat-sen University Cancer Center, *YNCH* Yunnan Cancer Hospital.

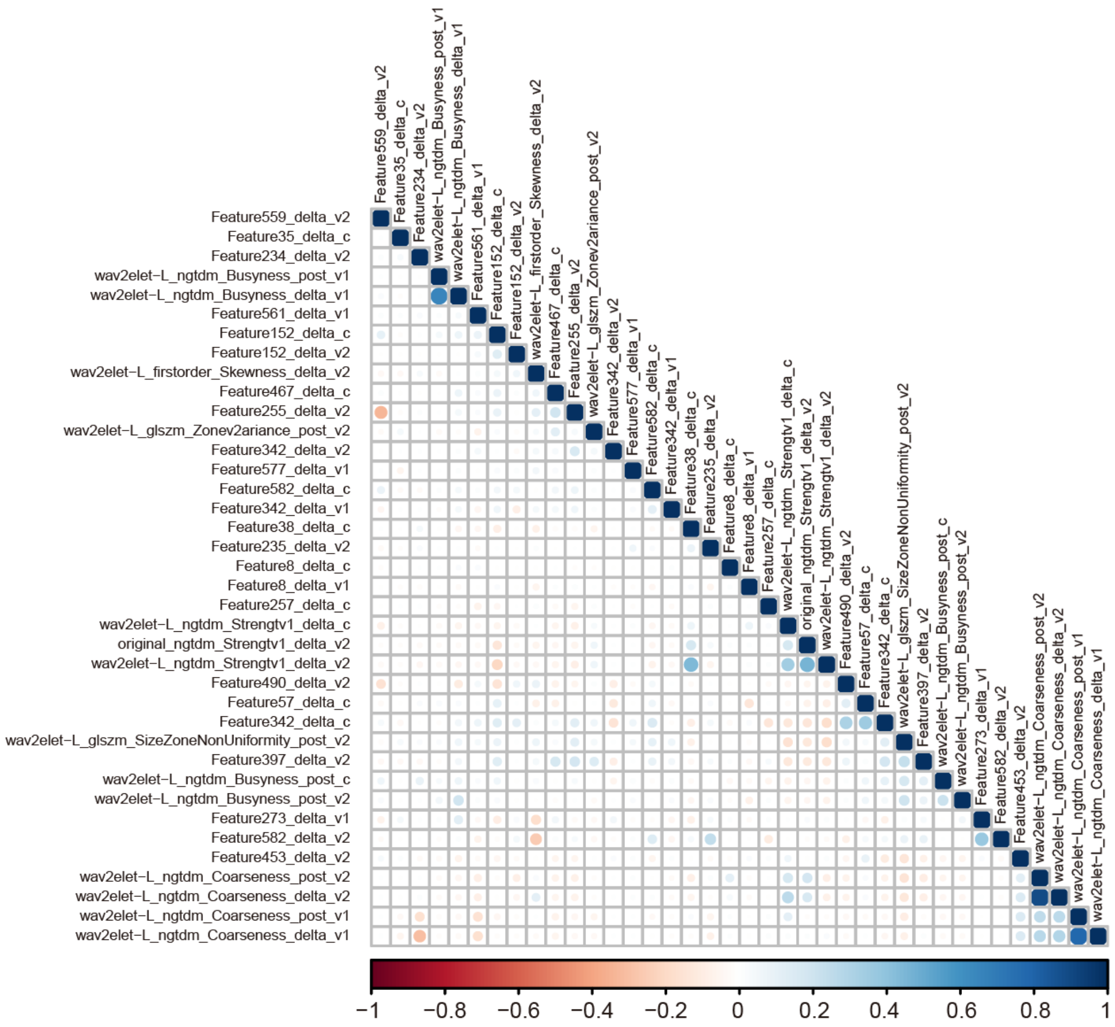

Supplementary Figure 2. Heatmap of deep learning and radiomics features expressions in the entire cohort. The bar represents Pearson. The features prefixed with "feature" correspond to those derived from deep learning methodologies, whereas the remaining features are indicative of radiomic characteristics.

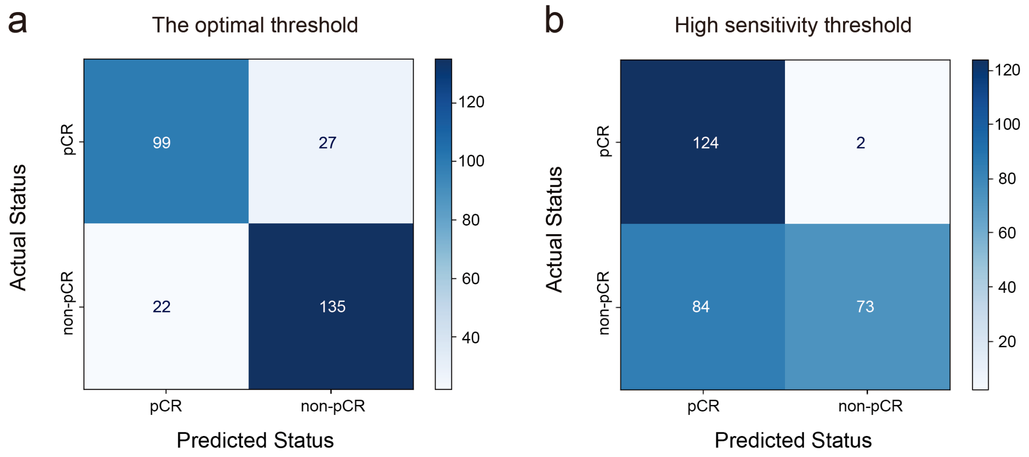

Supplementary Figure 3. Visualization of the confusion matrices for the BCRP model with different thresholds. (a) The optimal threshold which based on the maximum Youden index. **(b)** High sensitivity threshold. *pCR* pathological complete response.

Supplementary Table 1. Selected features for the Rad model construction.

post-NACT features (n = 7)	Delta features (n = 7)
wavelet-L_ngtdm_Busyness_post_color	wavelet-L_ngtdm_Strength_delta_ color
wavelet-L_ngtdm_Busyness_post_view1	wavelet-L_ngtdm_Busyness_delta_ view1
wavelet-L_ngtdm_Coarseness_post_ view1	wavelet-L_ngtdm_Coarseness_delta_ view1
wavelet-L_glszm_SizeZoneNonUniformity_post_ view2	original_ngtdm_Strength_delta_ view2
wavelet-L_glszm_ZoneVariance_post_ view2	wavelet-L_firstorder_Skewness_delta_ view2
wavelet-L_ngtdm_Busyness_post_ view2	wavelet-L_ngtdm_Coarseness_delta_ view2
wavelet-L_ngtdm_Coarseness_post_ view2	wavelet-L_ngtdm_Strength_delta_ view2

Delta features were obtained by analyzing the changes from pre-NACT to post-NACT. *NACT* neoadjuvant chemotherapy.

Supplementary Table 2 Comparison of model performances in the training and external test cohorts.

Compare models	Methods					
	Delong test	*P* value	NRI	*P* value	IDI	*P* value
**Training cohort**						
DL vs. Image	0.02	0.323	1.68	<0.001*	1.01	<0.001*
Rad vs. Image	0.06	0.005*	1.23	<0.001*	0.34	<0.001*
DL vs. BCRP	0.06	<0.001*	1.74	<0.001*	0.01	<0.001*
Rad vs. BCRP	0.10	<0.001*	1.44	<0.001*	0.34	<0.001*
Clinic vs. BCRP	0.28	<0.001*	2.00	<0.001*	0.90	<0.001*
BR vs. BCRP	0.32	<0.001*	2.00	<0.001*	0.94	<0.001*
**External test cohort**						
DL vs. Image	0.04	0.315	0.18	0.390	0.03	0.650
Rad vs. Image	0.08	0.020*	0.62	0.001*	0.10	0.151
DL vs. BCRP	0.19	0.005*	0.07	0.738	0.01	0.912
Rad vs. BCRP	0.11	0.010*	0.38	0.059	0.07	0.305
Clinic vs. BCRP	0.29	<0.001*	0.63	<0.001*	0.28	<0.001*
BR vs. BCRP	0.26	<0.001*	0.71	<0.001*	0.29	<0.001*

Data is metric values. An asterisk indicates a significant *P* value. *NRI* net reclassification improvement index, *IDI* integrated discrimination improvement.

Supplementary Table 3. Performance of the BCRP with high sensitivity threshold.

	Accuracy	Sensitivity	Specificity	PPV	NPV
Training cohort	0.78	0.99	0.55	0.71	0.98
External test cohort	0.56	0.97	0.37	0.42	0.97

PPV positive predictive value, *NPV* negative predictive value.

Supplementary Table 4. Multivariable Cox regression analysis of variables for event-free survival in total cohort.

Variables	Hazard ratio (95% CI)	*P* value
N stage		
N0	Ref	
N1	1.83 (0.81-4.12)	0.145
N2	3.19 (1.36-7.50)	0.008*
N3	5.31 (2.12-13.34)	<0.001*
Platinum		
No	Ref	
Yes	2.07 (1.16-3.71)	0.014*
BCRP		
Low score	Ref	
High score	0.28 (0.14-0.55)	<0.001*

An asterisk indicates a significant *P* value. *CI* confidence interval.
